# An evaluation of the clinician-facing research dashboards from the Toronto Adolescent and Youth (TAY) Cohort Study in mental health care

**DOI:** 10.1371/journal.pmen.0000605

**Published:** 2026-05-08

**Authors:** Sarah Kuburi, George Foussias, Julia Davies, Alicia Nunez Segovia, Jimmy Wong, Stephanie Ameis, Nicole Kozloff, Lena C. Quilty, Erin Dickie, Daniel Felsky, Benjamin I. Goldstein, Lisa D. Hawke, Alexia Polillo, Yuliya S. Nikolova, Wei Wang, TAY Cohort Study Team, Aristotle Voineskos, Kristin Cleverley

**Affiliations:** 1 Department of Applied Psychology and Human Development, Ontario Institute for Studies in Education, University of Toronto, Toronto, Ontario, Canada; 2 Campbell Centre for Mental Health Research Institute, Centre for Addiction and Mental Health, Toronto, Ontario, Canada; 3 Department of Psychiatry, Faculty of Medicine, University of Toronto, Toronto, Ontario, Canada; 4 Institute of Medical Science, University of Toronto, Toronto, Ontario, Canada; 5 Lawrence Bloomberg Faculty of Nursing, University of Toronto, Toronto, Ontario, Canada; 6 Institute of Health Policy, Management and Evaluation, University of Toronto, Toronto, Ontario, Canada; 7 Department of Psychological Clinical Science, University of Toronto Scarborough, Scarborough, Ontario, Canada; Instituto Federal do Maranhão: Instituto Federal de Educacao Ciencia e Tecnologia do Maranhão, BRAZIL

## Abstract

Dashboards that integrate patient research data for clinical use can streamline information sharing and support clinical care, yet their application in mental health care is underexplored. This study evaluated the clinician-facing dashboards developed from the Toronto Adolescent & Youth (TAY) Cohort Study at the Centre for Addiction and Mental Health (CAMH), guided by the Reach, Effectiveness, Adoption, Implementation, and Maintenance (RE-AIM) framework. Dashboard reach was assessed through quantitative analyses, while semi-structured interviews with seven clinicians were conducted to explore perceptions of effectiveness, adoption, implementation, and maintenance. Quantitative analyses demonstrate that dashboards are completed for 69% of participants. Following the initial implementation phase, the average time from participant consent to dashboard completion decreases to six months. Qualitative findings suggest that dashboards can serve as a supplementary information source that may aid in supporting clinical decision-making and the integration of patient-reported research data into care. Clinicians also identified areas for improvement, including delays in dashboard completion, difficulty locating dashboards, and inefficient dashboard completion notifications. Clinicians suggested that addressing these areas through improved accessibility, timely data availability, and aligned communication strategies can increase uptake and sustainability. These findings emphasize the value of engaging end users and conducting ongoing evaluation to optimize the integration of research data into clinical workflows using dashboards to enhance mental health care. Future research should further examine factors influencing dashboard use across mental health care settings.

## Introduction

Visual summaries of patient data can enhance diagnostic accuracy and support clinical decision-making, ultimately contributing to improved healthcare quality [1,2]. Dashboards exemplify this approach by simplifying complex patient information into accessible formats such as bar graphs, pie charts, and intuitive color-coding [3]. Information commonly displayed in dashboards includes clinical summaries, laboratory results, progress tracking, and research assessment outcomes [4]. Compared to conventional records entered into electronic medical record (EMR) systems, dashboards integrated within EMR systems provide a more streamlined presentation of these critical metrics [5]. Dashboard use has been associated with improved care processes, including reduced time spent on data gathering, lower cognitive load, and enhanced communication [6,7]. By improving the interpretability of clinically relevant information, dashboards can also facilitate shared decision-making with patients [8]. However, their effectiveness depends on several factors, such as usability, ease of access, organizational support, user roles, digital literacy, and the willingness of clinicians to modify existing workflows [9–12]. These factors also vary across clinical settings, underscoring the need for context-specific design and evaluation to support effective implementation and clinical value.

Despite widespread implementation and evaluation of dashboards in physical health care settings, including disease-specific units such as neurology, oncology, respirology, as well as in primary care [2,9], their use within mental health services remain limited. One study conducted in an inpatient adult mental health setting found that clinicians perceived dashboards as useful for rapidly accessing large amounts of clinically relevant information, though their effectiveness depended on the clarity of data presentation [13]. Another study involving mental health professionals working with elderly inpatients reported that dashboards improved information access, communication and information sharing, and staff awareness, but also noted challenges such as limited staff access, inaccurate data, and increased workload [14]. Further research is needed to understand the impact and usability of dashboards in mental health care, particularly their capacity to support service delivery in outpatient contexts and among younger populations such as adolescents and youth.

Previous research on dashboards in healthcare has increasingly drawn on implementation science to guide comprehensive evaluations [15,16]. Implementation science focuses on methods that facilitate the integration, adoption, and sustainability of evidence-based practices across various settings, while also developing strategies, informed by context, for improving the speed, extent, and quality of uptake [17,18]. This approach is particularly useful for evaluating dashboards, as it systematically addresses how these tools are implemented, adopted, and sustained in routine healthcare environments. A commonly applied framework within this field is the Reach, Effectiveness, Adoption, Implementation, and Maintenance (RE-AIM) framework, which guides the evaluation of programs according to these five categories [19]. Applying RE-AIM facilitates a comprehensive assessment of a dashboard’s impact by evaluating its uptake among intended users, fidelity of implementation, effectiveness in improving outcomes, and long-term sustainability, thereby identifying opportunities for optimization [20].

The Toronto Adolescent and Youth (TAY) Cohort Study is a five-year longitudinal investigation of 1,500 participants aged 11–24 years, conducted at the Centre for Addiction and Mental Health (CAMH), an academic health sciences centre and Canada’s largest research and teaching mental health hospital. The TAY Cohort Study examines developmental trajectories of mental health and functioning and identifies risk and protective factors among adolescents and youth seeking mental health care [21–23]. Clinician-facing dashboards developed from this study present key research findings from participants’ baseline mental health assessments, including a structured diagnostic interview, other interviewer-administered diagnostic tools, cognitive tests, and self-report measures. With participant consent, study data can be shared with clinicians through dashboards completed and uploaded to the patient’s EMR to support their care. To promote successful integration and uptake of research data into clinical practice, clinicians were actively engaged in the co-design of these dashboards. In addition to the clinician-facing dashboards, the TAY Cohort Study also developed a participant-facing dashboard to display cognitive testing results, which were not the focus of this evaluation.

Although these dashboards are in use, their implementation, effectiveness, and sustainability in leveraging research data to support patient care have not been systematically evaluated. This study addresses this gap by applying the RE-AIM framework to evaluate the reach, effectiveness, adoption, implementation, and maintenance of the TAY Cohort Study clinician-facing dashboards. Specifically, it seeks to answer the question: *How do clinician-facing dashboards developed from the TAY Cohort Study perform across the five RE-AIM categories in a mental health care setting?* Understanding how the TAY Cohort Study clinician-facing dashboards function are essential for optimizing the dashboard’s potential to enhance mental health service delivery by integrating research data into clinical practice.

## Materials and methods

### Ethics

This study received approval from the organization’s Research Ethics Board (REB #2024/138) before initiation and prior to participation, all individuals provided written informed consent.

### Study design

This study follows the *Guidance for Publishing Qualitative Research in Informatics* checklist to ensure transparency and comprehensive reporting [24]. S1 Table in the Supporting Information Files provides the checklist along with the location of each element within the manuscript.

This study adopted a mixed methods approach, primarily qualitative in nature, with quantitative data used to assess feasibility. The study was guided by the RE-AIM framework, which includes five categories: Reach, Effectiveness, Adoption, Implementation, and Maintenance [19]. Reach was assessed quantitatively by measuring the proportion of TAY participants who consented to share their data with clinicians, serving as a proxy for clinician access to the dashboards. This analysis utilized existing routinely collected data from the TAY Cohort Study to confirm that clinicians had sufficient access to the dashboards, thereby establishing the feasibility of conducting the qualitative component of the study.

The remaining RE-AIM categories were evaluated using qualitative data from clinician interviews. Effectiveness was assessed at the clinician level to explore the dashboards’ perceived impact on decision-making and care processes. Adoption explored the extent to which clinicians integrated dashboards into their workflows and the factors influencing uptake. Implementation considered the fidelity and consistency of dashboard delivery as intended, based on clinicians’ experiences. Maintenance was assessed through clinicians’ perceptions of whether dashboards could be sustained and incorporated into routine practice over time. Due to limitations in system analytics and the focus on clinician experiences rather than patients, a gap currently identified in the literature, objective, patient-level, and indicator-based metrics were not included in this evaluation [25].

### Setting and timeline

The present evaluation was conducted within the Child, Youth, and Family (CYF) services program, which encompasses inpatient and outpatient mental health care for children, youth, and their families. While individual clinics may serve different populations, the program overall treats individuals aged five to the mid- to late twenties. Care ranges from consultation to short- and long-term follow-up, without standardized criteria for ongoing care. Patients and/or their caregivers within the CYF services program may be invited to participate in the TAY Cohort Study (https://www.taycohort.ca/), and those who consent to share their results from the study via dashboards allow clinicians to access this information through the EMR system. Patients may be referred to the study at any point in their care timeline, for example during their initial intake visit or later while receiving ongoing care. The timeline from consent to dashboard availability involves several steps. After consent, participants complete diagnostic assessments and self-report measures, which take approximately three to four hours in total. Research staff then present findings at consensus meetings with senior clinicians, held to review assessment results and reach agreement on diagnoses. Once consensus is reached, dashboards are populated with diagnostic assessment findings and self-report measures and uploaded to the general documentation section of the EMR system for each patient. For an example of the TAY Cohort Study dashboard populated with mock data, please contact the corresponding author. The patient’s primary clinicians (i.e., psychologist or psychiatrist) are notified via email to their organizational account when a dashboard has been completed for their patient; however, all clinicians who are part of the patient’s care team can access the TAY Cohort Study dashboard through the EMR system. Although the TAY Cohort Study is longitudinal, dashboards are currently developed only for baseline assessments. For consistency, within the present manuscript, patients who chose to participate in the TAY Cohort Study will hereafter be referred to only as participants, with the intention of capturing their dual role as both patients in the CYF services program and TAY Cohort Study participants.

### Participants and procedures

Routinely collected data were first requested from the TAY Cohort Study team to confirm clinician access to the dashboards by determining the proportion of TAY participants who consented to share their data with clinicians, thereby establishing feasibility for the qualitative component. Eligible participants were clinicians in the CYF services program who had access to, or prior experience using the dashboards. Recruitment occurred via email and during clinical team meetings, where a research team member introduced the study. Each clinician was contacted three times over a period of three to four weeks to accommodate busy schedules and increase the likelihood of participation. Interested clinicians scheduled a session with a research analyst to provide informed consent and complete a 30-minute semi-structured qualitative interview, conducted in person or via videoconference. Clinicians were recruited and interviewed between April 2, 2025, and May 26, 2025. As the TAY Cohort Study is ongoing and continues to actively recruit, this evaluation includes data from participants enrolled between May 4, 2021, and March 12, 2025 (1,053 out of a target of 1,500). At the time of this data cut, approximately 63 clinicians including psychologist, psychiatrists, nurses, and social workers, had access to the dashboards.

### Measures

Quantitative data routinely collected through the TAY Cohort Study, specifically participant consent and dashboard availability, were extracted to assess dashboard reach. These data included: (1) the date of the participant’s consent discussion; (2) whether consent was provided to share dashboards with clinicians; and (3) the date of dashboard completion. A semi-structured interview guide, informed by four of the five RE-AIM framework categories, was developed to explore clinicians’ perceptions of the dashboards, with example questions presented in Table 1. The interview guide was co-developed with the TAY Cohort Study’s youth advisory group to ensure their perspectives on the use of their clinical data were fully incorporated. Youth input focused on understanding what they want to know about how clinicians interact with their research data and how this may affect their care. Their feedback was actively integrated to address the questions and concerns most important to them. In addition to the interview guide, clinician participants provided basic information about their professional designation, years of experience, and the populations with whom they typically work.

**Table 1:** RE-AIM framework definitions, data sources, example questions, and subcategories.

RE-AIM Framework Categories	Definition	Data Sources	Examples of Semi-Structured Interview Questions	Generated Subcategories
Reach	Proportion of participants who consented to share their research data	Quantitative, consent and completion data	N/A	N/A
Effectiveness	Measures the dashboard’s impact on key outcomes, such as clinician’s perspectives on decision-making and care processes	Qualitative, semi-structured interview	Can you share any instances where the dashboards have impacted your clinical decision-making processes?	Dashboards as a valuable supplement to patient information that supports decision-making, reduces frustration, and fosters collaborative care
Adoption	Assesses how clinicians integrate the dashboards into clinical workflows, including factors affecting uptake	Qualitative, semi-structured interview	Are there any strategies or resources that you believe could enhance adoption of the dashboards among clinicians?	Clinicians’ lack of awareness of the dashboards and their perspectives on how to increase adoption
Implementation	Examines fidelity and consistency of dashboard delivery as intended	Qualitative, semi-structured interview	How effective are the dashboards in providing relevant and timely information for care?	Clinicians’ perceptions of the dashboard’s design and formatting Clinicians’ practical suggestions for improving its integration into clinical workflows
Maintenance	Considers the extent to which the dashboards are sustained and integrated into routine clinical practice over time	Qualitative, semi-structured interview	Are there any ongoing support or training needs that you believe are essential for maintaining the effectiveness of the dashboards?	Leveraging existing TAY Cohort data for ongoing clinical use Enhancing workflow integration to support long-term use

### Data analytic strategy

Descriptive statistics summarized dashboard reach, using three metrics: (1) the proportion of participants who consented to share their results with clinicians; (2) among those who consented, the proportion of dashboards completed between May 4, 2021, and March 12, 2025; and (3) the average time from participant consent to dashboard completion. To assess potential effects of time on completion duration, reflecting early implementation challenges, average completion times were compared between the first and second halves of participants who had their dashboards completed. Quantitative data were analyzed using SPSS version 29.0 [26].

Semi-structured qualitative interviews were audio-recorded and transcribed verbatim. Data were analyzed concurrently with collection using directed content analysis, a commonly used method in healthcare research that facilitates examination of data through predefined concepts, in this case the RE-AIM framework [27]. Conducting analysis alongside data collection allowed early insights to inform subsequent interviews, enabling exploration of emerging subcategories and refinement of the inquiry based on observed patterns. Data collection ceased once insights from clinician interviews began to sound redundant and no new subcategories were emerging [28]. NVivo software version 14.0 was used to manage and organize the qualitative data [29].

Each transcript was coded according to the RE-AIM framework categories, and illustrative quotations were selected to highlight key subcategories. Subcategories within each RE-AIM category were developed deductively from the interview data and refined through ongoing discussions among the researchers, with overlapping or redundant categories merged or eliminated to create a clear coding structure. To enhance the trustworthiness of the findings, the first author (SK) led the coding, analysis, and interpretation, regularly reviewing decisions with the third (JD) and last (KC) authors [30]. These researchers collaboratively discussed interpretations, resolved discrepancies, and reached consensus on the definitions of each subcategory within the RE-AIM framework. Representative quotations are provided alongside each subcategory to support the validity of the findings [31].

## Results

### Sample description

Seven clinicians participated in this study, resulting in a response rate of just over 11%. Clinicians represented disciplines such as medicine (psychiatry), psychology, and nursing, with experience ranging from a couple of months to over 20 years. The characteristics of the recruited sample largely resemble those of the broader clinician population who had access to and/or used the dashboards. The populations with whom they worked included adolescents and youth aged 11–24 with autism, mood and anxiety disorders, substance use disorders, and concurrent disorders, across inpatient and outpatient settings.

### Primary findings

**Fig 1:**
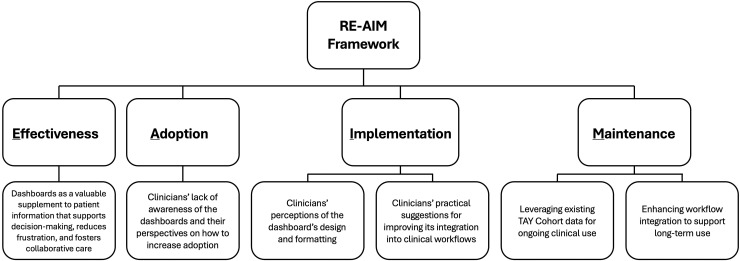
Visual representation of the subcategories by each RE-AIM category.

Quantitative data informing the Reach category, along with subcategories derived from interview transcripts informing the other RE-AIM categories, are presented below. Subcategories are organized by RE-AIM category and illustrated with representative quotes. Table 1 summarizes the subcategories, their data sources, and example interview questions, and Fig 1 provides a visual representation of the subcategories.

#### Reach.

Out of a final enrollment target of 1,500 participants for the TAY Cohort Study, 1,053 had been recruited as of March 12, 2025. Descriptive analyses indicated that 1,024 participants (97.2%) consented to share their study data with clinicians. Among these, 709 participants (69.2%) had their dashboards completed and uploaded into the EMR system, by the same date. The mean time from consent to dashboard completion was 251 days (SD = 151.92), or approximately 8.2 months, with a range of 37–794 days. Dashboards that were not completed or uploaded resulted from delays in scheduling diagnostic assessment interviews, delays in holding consensus meetings, or delays in populating dashboards after these steps were completed.

**Fig 2:**
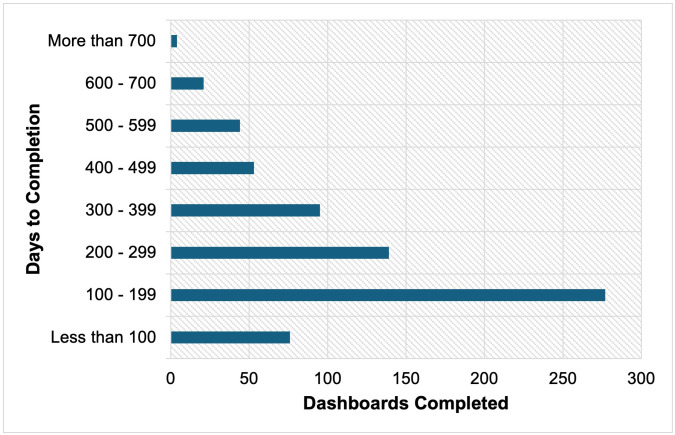
Dashboard completion per 100-day interval.

To account for potential delays during the initial implementation phase, the time from consent to dashboard completion was compared between the first half of participants (n = 354 of 709) and the subsequent half (n = 355 of 709) whose dashboards had already been completed and uploaded into the EMR system. Among the first half, the mean time from consent to completion was 320 days (SD = 170.88), approximately 10.5 months, with a range of 47–794 days. In the subsequent half, the mean time decreased to 182 days (SD = 86.35), approximately 6 months, with a range of 37–526 days. A breakdown of dashboard completion per 100-day interval across the TAY Cohort Study period is presented in Fig 2.

#### Effectiveness.

Within the Effectiveness category, one subcategory was generated: dashboards as a valuable supplementary source of information that supports clinical decision-making, reduces participant frustration, and promotes collaborative care.

Clinicians described the dashboard as a useful secondary source of information, particularly when first working with a client or conducting initial assessments: “For me, it’s so helpful to have that added information, especially when I’m starting to engage with a client. To be able to see that and where things are at more recently versus when they were referred is super useful.” (Clinician 4)

Clinicians also noted that, although the dashboard may not consistently drive clinical decisions, it can serve as an important reference when specific information is sought: “The dashboard, whenever it comes in, is a source of information. It doesn’t directly inform clinicians’ decision-making unless it’s specifically referenced. For example, if a clinician wants to know, ‘What are the assessment findings [of a specific measure] that could guide my next suggestion?’ then yes, the TAY dashboard can be very helpful.” (Clinician 1)

Participants also emphasized how dashboards may help alleviate participant frustration associated with repeatedly sharing the same information across services: “I just believe that if kids are taking the time to fill out this information, it should influence their care. Kids often say, ‘I already told someone that. Why do I have to do it again? Why fill out this questionnaire again?’ Repetition like that can feel frustrating and make them lose faith in the system.” (Clinician 5)

Finally, clinicians highlighted the potential for dashboards to support recovery-oriented care by fostering shared understanding between clinicians and participants: “There are participants who just adore it. You give them information, you screen, you provide questionnaires. The more you invest in them and reflect with them, the more it becomes part of their journey to recovery.” (Clinician 7)

#### Adoption.

Within the Adoption category of the RE-AIM framework, one subcategory was also generated: clinicians’ lack of awareness of the dashboards and their perspectives on how to increase adoption.

Many clinicians reported infrequent use of the dashboards, often attributing this to a lack of awareness. For example, some stated simply, “Yeah, I don’t use them” (Clinician 2) and “Probably not as often as I should.” (Clinician 7) Another clinician shared: “I think I found out about it the first time just by accident. I was in the chart, saw something, clicked on it, and realized it was the dashboard. I remember thinking, ‘That would have been good to know.’” (Clinician 3) One clinician also noted that the current study was their first exposure to the dashboard: “Probably from this study. I hadn’t heard about it at all before, so I was like, ‘Wait, there’s a dashboard?’” (Clinician 2)

Although not all clinicians appeared to have been oriented to the dashboard, some recalled brief information shared by the TAY Cohort Study team during their clinical team meetings, which may not have effectively engaged staff. One reflected: “There might have been like a five-minute meeting just to kind of look at the dashboard and let people know it’s there and give an example of one, but I don’t remember any kind of formal training around it.” (Clinician 6) Another added: “I think I forgot the year when it took place, but I think others have brought that to the meetings.” (Clinician 1)

Consequently, during the interviews, clinicians were asked what might enhance their adoption of the dashboards. They emphasized that easily accessible and visually engaging resources could improve awareness and use. Specifically, some suggested tools such as infographics or quick-reference guides would be helpful given their limited time: “Even something like an infographic about what’s included in the dashboard and how it could be used, like a tip sheet type of idea.” (Clinician 1)

“Maybe there could be something available through the TAY website, like a resource or a link for clinicians with a description of the TAY dashboard, something they could review on their own time.” (Clinician 4)

#### Implementation.

Within the Implementation category of the RE-AIM framework, two subcategories were generated: clinicians’ perceptions of the dashboard’s design and formatting, and their practical suggestions for improving its integration into clinical workflows.

Clinicians highlighted the dashboard’s clear organization and intuitive layout, which facilitated rapid and effective interpretation of participant information. One clinician noted, “It’s well organized and visually easy to follow.” (Clinician 4) Another added: “The organization, coloring, and contrast are all quite clear. It provides a quick visual of where things are at, by domain. I’m actually looking at a dashboard now and I like it.” (Clinician 1) Another clinician appreciated the visual presentation of the data: *“*I really like, under the psychosis screening, how the score is represented. That’s visually easy to see. The substance scale is useful too. There’s a lot of information. It would probably take me a little bit of getting used to before I find it really helpful, but it’s cool. That’s a lot of information.*”* (Clinician 5)

To further enhance implementation, clinicians offered suggestions aimed at streamlining dashboard use and better embedding it into existing workflows. These included optimizing the timing of dashboard availability, improving ease of access within the EMR system, and tailoring notification methods.

For example, some clinicians highlighted the potential to increase dashboard usefulness by minimizing delays in dashboard availability. One shared, “The delay. Ideally it could come earlier. Something that arrives nine months later isn’t that useful.” (Clinician 3) Another pointed out that earlier access could enhance clinical relevance: “By the time I get the clinical dashboards, I’ve already formed my impression of what’s going on.” (Clinician 6) Others noted that timely information is particularly critical given the rapid development of adolescent and youth participants: “Teens develop so quickly, so even a few months might be significant in terms of how seriously you take the information.” (Clinician 5)

Clinicians also suggested that improving the dashboard’s placement within the EMR system would increase accessibility and usability. As one clinician explained, “I’m actually looking into a participant’s file that I believe has a dashboard somewhere, but it’s hard to find through the EMR system.” (Clinician 1) Another added: “It’s more about making them easier to find in the EMR system.” (Clinician 6)

Regarding notifications, clinicians preferred integrating notifications directly into the EMR messaging system rather than using email, which they found less effective for their workflow. One clinician noted, “I have never stopped what I was doing because an email came in. I’m in the middle of something else. I’m not going to stop, load the EMR system, search the MRN [Medical Record Number, a unique patient identifier used to access medical information within an EMR)], find the dashboard, and then try to figure out if it’s still relevant months later.” (Clinician 6) Another suggested, “Clinicians check the message system in the EMR. So, if the dashboard became a message sent through that system, it would be more likely to get our attention.” (Clinician 1)

To reduce completion delays, clinicians recommended prioritizing participant self-report measures that can be completed promptly, noting that interviewer-administered diagnostic tools often require time-consuming consensus meetings to confirm diagnoses. One clinician explained, “The diagnostic tools just cause more delays than they’re worth. The self-report data is still helpful and can be uploaded immediately.” (Clinician 6)

Finally, to enhance dashboard accessibility, clinicians suggested aligning the dashboard location within the EMR system with that of other dashboards. One clinician mentioned: “All the other dashboards are in a separate file, so it is very easy for clinicians from other programs to log into the EMR system and access their dashboards directly. Instead of searching through documentation files, it would be helpful if this TAY Cohort dashboard were also located in the dedicated dashboard section.” (Clinician 7)

#### Maintenance.

Within the Maintenance category of the RE-AIM framework, two subcategories were generated: clinicians emphasized the value of continuing to leverage existing data from the TAY Cohort Study and identified opportunities to strengthen implementation to support long-term integration into clinical workflows.

Clinicians recognized that ongoing assessments already conducted through the TAY Cohort Study offer a valuable foundation for generating dashboards that could inform care. As one clinician explained: “Well, I mean, if you’re doing the assessments as part of the study, those are already happening. So, it makes sense that if there’s a youth we’re still seeing, we should get that data. That would be helpful to have.” (Clinician 3) Another clinician reflected on the alignment between dashboard use and measurement-based care: “From a values perspective, I think that information should impact clinical care. I want to be part of a program that supports integrating information, for sure.” (Clinician 5)

While clinicians acknowledged the future potential of dashboards, they also noted areas where streamlining processes could enhance their utility. For example, one clinician shared: “I really think this could be useful. It’s just that there may be too many steps to access.” (Clinician 1) Another clinician also explained: “Yeah, I think it has a lot of potential. It’s just about maximizing the efficiency.” (Clinician 4) This clinician further expanded, “100% I feel like this is something that could have so much value if it were designed in a way that’s compatible with the EMR system.” (Clinician 4)

## Discussion

Guided by the RE-AIM framework [19], this study employed a mixed-methods design that was primarily qualitative, with quantitative data used to assess feasibility. The objective of this evaluation was to assess the reach, adoption, implementation, effectiveness, and maintenance of the clinician-facing dashboards developed for the TAY Cohort Study; a five-year research project aimed at identifying markers of longitudinal mental health and functional outcomes among adolescents and youth receiving care at an academic mental health hospital. Although prior research has examined dashboards in clinical care settings, there has been limited evaluation into their use within mental health services. This gap underscores the need to better understand the impact, usability, and sustainability of dashboards across diverse mental health care contexts. The RE-AIM framework provided a structured approach for this evaluation, enabling the study to examine not only whether the dashboards were effective but also how well they were adopted, implemented, and sustained in a real-world clinical setting. The findings of this evaluation provide critical insights into clinician engagement with the dashboards, their practical application and impact within clinical settings, and offer realistic suggestions to enhance the integration of research data into dashboards to leverage this data to inform and improve mental health care.

An important finding from this evaluation is that 97.2% of TAY Cohort Study participants enrolled between May 4, 2021, and March 12, 2025, consented to share their research data with clinicians. This demonstrates that participants engaged in research value having their data inform their mental health care and clinicians’ decision-making, supporting previous work showing that individuals appreciate research with direct clinical implications [32]. Clinicians highlighted the value of using research data to develop dashboards, noting that they provided a helpful additional source of information. Consistent with previous research on dashboard integration within mental health care settings [13,14], clinicians in the present study suggested that the dashboards aided in decision-making in clinical care and provided a practical way to incorporate patient-reported data into care. Clinicians reported the dashboards were especially useful early in a participant’s care timeline, as they provided important information at the outset. Advance access to patient self-reported information may improve efficiency, support patient engagement, and reduce follow-up work after the visit [33]. Clinicians also appreciated the dashboards’ design and formatting, consistent with evidence that user-friendly dashboards promote positive adoption in healthcare [34]. These findings demonstrate the value of clinical research collaborations and embedding research data directly into practice as a strategy to accelerate the translation of research findings into improvements in mental health care.

To enhance the usefulness and sustainability of dashboards presenting research data within clinical practice, clinicians identified several areas for improvement. Given the limited evaluation of dashboards in mental health care settings, identifying areas for improvement provides context-specific recommendations within these settings through consideration of the implementation challenges and suggested solutions, which may strengthen clinician engagement and support real-world integration of future dashboards in mental health care. Areas for improvement identified in this study included delays in dashboard completion, difficulty locating dashboards within the EMR system, and dashboard completion notification issues, such as missed alerts due to poor integration with existing workflows. Research indicates that for new tools to be effective and sustainable, they must be seamlessly integrated into existing clinical workflows and systems [35]. System-level solutions such as enhanced EMR integration strategies and personalized alerts tailored to individual clinician preferences may help resolve location and notification challenges identified in this study [36,37]. These challenges may have limited dashboard adoption and hindered the ability to fully evaluate their integration, effectiveness, and long-term sustainability within this study using the RE-AIM framework. Clinicians emphasized that addressing these issues through improved access, timely data availability, and enhanced communication strategies could support greater integration and more effective use of the dashboards in everyday clinical practice.

The present findings align with prior research emphasizing the importance of immediate and streamlined access to information to promote regular use [6]. Delays in dashboard completion, observed in both the quantitative and qualitative findings, affected user adoption and were a critical barrier to implementation. For example, some clinicians received dashboards after the information was no longer clinically relevant or after they had formed their clinical opinion. Although average completion times decreased between the first and second halves of participants, the latter group still experienced an average wait of approximately six months, suggesting that while dashboard completion times were initially long, they are improving over time. Nonetheless, these delays highlight the gap between participant consent and dashboard completion, limiting access to real-time data necessary for timely clinical decision-making [38]. These results highlight the importance of timely, accessible, and user-centered tools in mental health care and the need to align dashboard completion with clinical timelines to maximize impact [39]. This is particularly critical given the rapid developmental changes and life transitions experienced by adolescents and youth [40]. Additionally, given the longitudinal nature of the TAY Cohort Study, leveraging data from longitudinal studies to create timely dashboards that track changes over time may be especially valuable for supporting adolescents and youth as well as their clinicians during this critical stage of development.

In this evaluation, clinicians identified practical strategies to address key implementation challenges, including removing interviewer-administered assessments that contribute to delays in dashboard completion. Additionally, clinicians reported limited awareness of the dashboards and a lack of formal training to support their use. To address these gaps, clinicians suggested incorporating enhanced communication strategies, such as easy-to-access infographics and tip sheets, to improve awareness and facilitate integration into clinical workflows. Addressing these challenges may help dashboards better align with the principles of measurement-based care, which emphasize the routine collection and use of patient data to inform and improve treatment decisions [44]. These findings highlight the importance of actively involving clinicians not only in design and initial implementation phases but also in ongoing evaluation and refinement processes, as was done in this study, to support sustained integration into clinical workflows. Such engagement fosters clinician acceptance and active use of dashboards, both of which are critical for successful adoption into practice [45]. Despite this, end-user engagement in healthcare innovation remains inconsistent, as a literature review on dashboard development, implementation, and evaluation found that only half of studies involved end users during the design and implementation phases [42].

Although clinicians in this study expressed interest in the dashboards and recognized their potential benefits, existing challenges may have limited their consistent use. The implementation of the TAY Cohort Study dashboards are still being developed and refined within CYF services, and this evaluation aims to help optimize the dashboard process and provide a foundation for demonstrating their utility and sustainability. Improving implementation strategies is therefore essential to enhance dashboard effectiveness and long-term sustainability within mental health care settings. Implementation science frameworks, such as RE-AIM, offer a structured approach for evaluating new tools, improving the rigor and comparability of results, and generating more actionable evidence for practice [41]. Despite this, a systematic review of dashboard development, implementation, and evaluation in healthcare found that only about 20% of studies used a theory or framework to guide their evaluation [42]. Among those that did, studies applying frameworks such as RE-AIM to inform implementation-related research questions, as was done in the present study, have provided valuable insights for improving dashboard implementation in clinical settings from the perspectives of end users [43].

### Implications

This study underscores the importance of involving clinicians not only in the co-design and implementation of dashboards but also in their ongoing evaluation to maintain clinical relevance [46,47]. Equally important is the engagement of operational staff responsible for deployment and maintenance to ensure effective functioning. For example, this study identified delays between participant consent and dashboard completion. Although specific segments of the process could not be isolated, efforts are underway to reduce delays by streamlining workflows in collaboration with research staff. A key contributor to these delays is the need to confirm diagnostic assessment findings in consensus meetings, which typically occur one to two months after assessments. The organization is working with research staff to increase the frequency of presenting diagnostic findings at these meetings, with the goal of ensuring they occur within days of the assessment. This approach may reduce the timeline from participant consent to dashboard completion by one to two months. Alternatively, as suggested in this study, removing assessments that require confirmation through consensus meetings may also help reduce delays, since self-report assessments are immediately available for use in clinical contexts. These findings highlight that successful dashboard integration depends on both clinician engagement and coordinated staff contributions, emphasizing the importance of their involvement in evaluation initiatives.

Emerging implications of this study may also suggest the potential value of leadership roles such as Chief Medical Informatics Officers, who serve as liaisons between clinical practice and information technology within academic healthcare organizations [48], as these individuals and their teams can help prioritize and support dashboard implementation. Areas for improvement identified in this study, including enhancing dashboard visibility and developing clinician-requested alerts, may particularly benefit from their expertise and oversight. The organization in which this study was conducted has established a Digital Innovation Hub (https://www.camh.ca/en/science-and-research/digital-innovation-hub) to accelerate the design, development, implementation, and scaling of digital health innovations to support mental health and substance use care by fostering partnerships among researchers, clinicians, and industry partners. Although the present study did not leverage this resource, as it was established after the initiation of the TAY Cohort Study, future work could benefit from such collaboration. Healthcare organizations considering dashboard implementation may consider assessing whether comparable roles or resources exist, as they may be helpful in supporting successful implementation, enhancing effectiveness, and facilitating the integration of research data into routine clinical care.

The findings of this study may also emphasize the importance of pilot testing dashboard tools with representative clinicians within their workflows, rather than solely relying on co-design, before broader implementation. While co-design can ensure dashboards are visually appropriate, usability testing provides critical insights into practical challenges, such as difficulties locating dashboards or inefficient notification processes, that may impede adoption or reduce effectiveness. Prioritizing usability testing within real world clinical contexts enables early identification of workflow mismatches and ensures tools are both relevant and usable. Engaging clinicians early in this process may further enhance engagement, increases perceived utility, and supports long-term adoption [11,49,50]. Despite its importance, formative usability testing is reported even less frequently, with only 22% of studies incorporating this process [42]. Future dashboard development and evaluation should consider prioritizing usability testing to enhance dashboard quality, facilitate integration into clinical workflows, and support the translation of research data into routine clinical care, ultimately maximizing their impact on mental health care [51].

### Strengths and limitations

The present study has several strengths, including a novel contribution to an underexplored area, a theory-informed evaluation using the RE-AIM framework, a mixed methods design that integrated both qualitative and quantitative data, and incorporation of end-user experiences to address the study objectives. However, several important limitations should be considered when interpreting these findings. First, the sample included only seven clinicians, primarily psychologists and psychiatrists, with limited representation from nursing, resulting in a response rate of approximately 11%. Although multiple disciplines were represented, the small and self-selected sample may not fully capture the range of experiences with the dashboards within the CYF services program. In particular, perspectives from social workers, nurses, and other clinicians were underrepresented, which may limit the validity and transferability of these findings. Furthermore, while data collection ceased once interviews with participating clinicians began to show redundancy and no new subcategories emerged, additional perspectives from underrepresented professions may have yielded new or divergent insights or further corroborated the present findings. Second, due to data limitations, differences between specific segments of the dashboard completion timeline, including consent to diagnostic assessment, assessment to consensus meeting, and consensus meeting to dashboard availability, could not be examined. Additionally, due to data limitations within the EMR system, we were unable to determine the total number of clinicians who have used the TAY Cohort Study dashboards. Third, the effectiveness of the clinician-facing dashboards was evaluated solely at the clinician level using qualitative interviews. No objective or indicator-based data were incorporated to assess effectiveness, such as changes in clinical decision-making, participant outcomes, or service efficiency. Fourth, self-selection bias is a potential concern, as clinicians with stronger opinions, either positive or negative, may have been more likely to participate, whereas less engaged or neutral clinicians may have opted out, limiting perspective diversity [52]. Some participants also reported implementation challenges that affected dashboard use, limiting the ability to fully assess categories such as adoption, effectiveness, and sustainability due to their limited hands-on experience.

### Future directions

Further research is needed to explore these implementation issues in greater depth, ideally through the integration of multiple data sources (e.g., objective, indicator-based metrics) and methodological approaches. Research in this area should also seek to identify additional contextual factors that may hinder the uptake of clinician-facing research dashboards in clinical care, particularly given the limited evaluation of such dashboards in mental health settings [13]. It will also be important to examine the experiences of mental health professionals from other disciplines in their use of clinician-facing dashboards to identify potential differences in their experiences. Finally, subsequent evaluations may benefit from reassessing RE-AIM categories after initial implementation challenges have been addressed, as perceptions of reach, effectiveness, adoption, implementation, and maintenance may evolve over time. Attention should also be given to how barriers encountered within one category may influence or shape perceptions in other domains, given the interrelated nature of the RE-AIM framework.

### Conclusion

Guided by the RE-AIM framework, this study used a mixed-methods design that was primarily qualitative, supplemented by quantitative data, to evaluate the reach, implementation, adoption, effectiveness, and maintenance of clinician-facing dashboards developed from a large adolescent and youth mental health and functioning cohort study at a research and teaching hospital in Canada. Findings indicate that clinicians viewed the dashboards as a valuable supplementary source of information that may aid in supporting clinical decision-making and the integration of participant-reported data into care. Challenges such as delays in dashboard completion, difficulty locating dashboards, and misaligned notification processes limited adoption and may have hindered a full assessment of effectiveness, implementation, and sustainability. Clinicians recommended improving access, timely data availability, and communication strategies to enhance usability and uptake. These findings may suggest the importance of engaging end users, operational staff, and subject matter experts collaboratively, alongside usability testing and continuous evaluation, to support the effective integration of research data into clinical workflows and improve mental health care. Future research should explore factors affecting dashboard use in various mental health care settings and how challenges in one RE-AIM category may influence others.

## Supporting information

S1 Table Guidance for publishing qualitative research in informatics reporting checklist. (DOCX)

## References

[1] Park S, Bekemeier B, Flaxman A, Schultz M. Impact of data visualization on decision-making and its implications for public health practice: a systematic literature review. Inform Health Soc Care. 2022;47(2):175–93. doi: 10.1080/17538157.2021.1982949 34582297

[2] Bucalon B, Shaw T, Brown K, Kay J. State-of-the-art dashboards on clinical indicator data to support reflection on practice: scoping review. JMIR Med Inform. 2022;10(2):e32695. doi: 10.2196/32695 35156928 PMC8887640

[3] West VL, Borland D, Hammond WE. Innovative information visualization of electronic health record data: a systematic review. J Am Med Inform Assoc. 2015;22(2):330–9. doi: 10.1136/amiajnl-2014-002955 25336597 PMC4394966

[4] Hysong SJ, Yang C, Wong J, Knox MK, O’Mahen P, Petersen LA. Beyond information design: designing health care dashboards for evidence-driven decision-making. Appl Clin Inform. 2023;14(3):465–9. doi: 10.1055/a-2068-6699 37015343 PMC10266903

[5] Breton M, Gaboury I, Bordeleau F, Lamoureux-Lamarche C, Martin É, Deslauriers V, et al. Use of electronic medical record data to create a dashboard on access to primary care. Healthc Policy. 2023;18(4):72–88. doi: 10.12927/hcpol.2023.27092 37486814 PMC10370395

[6] Dowding D, Randell R, Gardner P, Fitzpatrick G, Dykes P, Favela J, et al. Dashboards for improving patient care: review of the literature. Int J Med Inform. 2015;84(2):87–100. doi: 10.1016/j.ijmedinf.2014.10.001 25453274

[7] Khairat SS, Dukkipati A, Lauria HA, Bice T, Travers D, Carson SS. The impact of visualization dashboards on quality of care and clinician satisfaction: integrative literature review. JMIR Hum Factors. 2018;5(2):e22. doi: 10.2196/humanfactors.9328 29853440 PMC6002673

[8] Perry LM, Mohindra NA, Coughlin A, Bedjeti K, Barnard C, Garcia SF, et al. Implementation of patient-reported outcome dashboards within the electronic health record to support shared decision-making in serious chronic illness. BMJ Open Qual. 2025;14(1):e002837. doi: 10.1136/bmjoq-2024-002837 39800390 PMC11752041

[9] Bersani K, Fuller TE, Garabedian P, Espares J, Mlaver E, Businger A, et al. Use, perceived usability, and barriers to implementation of a patient safety dashboard integrated within a vendor EHR. Appl Clin Inform. 2020;11(1):34–45. doi: 10.1055/s-0039-3402756 31940670 PMC6962088

[10] Bischof AY, Kuklinski D, Salvi I, Walker C, Vogel J, Geissler A. A collection of components to design clinical dashboards incorporating patient-reported outcome measures: qualitative study. J Med Internet Res. 2024;26:e55267. doi: 10.2196/55267 39357042 PMC11483256

[11] Gao G, Martin CL, Vaughan C, Markland A, Kelly U, Pathak N, et al. End users’ perceived engagement with clinical dashboards: a rapid review. Stud Health Technol Inform. 2024;310:1091–5. doi: 10.3233/SHTI231133 38269983 PMC12309167

[12] Patel SB, Iqbal FM, Lam K, Acharya A, Ashrafian H, Darzi A. Characterizing behaviors that influence the implementation of digital-based interventions in health care: systematic review. J Med Internet Res. 2025;27:e56711. doi: 10.2196/56711 40505130 PMC12203025

[13] Phull J, Hall J. Clinical dashboards and their use in an adult mental health inpatient setting, a pilot study. Clin Gov Int J. 2015;20(4):199–207. doi: 10.1108/CGIJ-06-2015-0019

[14] Daley K, Richardson J, James I, Chambers A, Corbett D. Clinical dashboard: use in older adult mental health wards. Psychiatrist. 2013;37(3):85–8. doi: 10.1192/pb.bp.111.035899

[15] Barnes GD, Sippola E, Ranusch A, Takamine L, Lanham M, Dorsch M, et al. Implementing an electronic health record dashboard for safe anticoagulant management: learning from qualitative interviews with existing and potential users to develop an implementation process. Implement Sci Commun. 2022;3(1):10. doi: 10.1186/s43058-022-00262-w 35109916 PMC8812192

[16] Dunn S, Sprague AE, Grimshaw JM, Graham ID, Taljaard M, Fell D, et al. A mixed methods evaluation of the maternal-newborn dashboard in Ontario: dashboard attributes, contextual factors, and facilitators and barriers to use: a study protocol. Implement Sci. 2016;11:59. doi: 10.1186/s13012-016-0427-1 27142655 PMC4855363

[17] Bauer MS, Damschroder L, Hagedorn H, Smith J, Kilbourne AM. An introduction to implementation science for the non-specialist. BMC Psychol. 2015;3(1):32. doi: 10.1186/s40359-015-0089-9 26376626 PMC4573926

[18] Handley MA, Gorukanti A, Cattamanchi A. Strategies for implementing implementation science: a methodological overview. Emerg Med J. 2016;33(9):660–4. doi: 10.1136/emermed-2015-205461 26893401 PMC8011054

[19] Glasgow RE, Vogt TM, Boles SM. Evaluating the public health impact of health promotion interventions: the RE-AIM framework. Am J Public Health. 1999;89(9):1322–7. doi: 10.2105/ajph.89.9.1322 10474547 PMC1508772

[20] Kwan BM, McGinnes HL, Ory MG, Estabrooks PA, Waxmonsky JA, Glasgow RE. RE-AIM in the real world: use of the RE-AIM framework for program planning and evaluation in clinical and community settings. Front Public Health. 2019;7:345. doi: 10.3389/fpubh.2019.00345 31824911 PMC6883916

[21] Cleverley K, Foussias G, Ameis SH, Courtney DB, Goldstein BI, Hawke LD, et al. The Toronto adolescent and youth cohort study: study design and early data related to psychosis spectrum symptoms, functioning, and suicidality. Biol Psychiatry Cogn Neurosci Neuroimaging. 2024;9(3):253–64. doi: 10.1016/j.bpsc.2023.10.011 37979943

[22] Dickie EW, Ameis SH, Boileau I, Diaconescu AO, Felsky D, Goldstein BI, et al. Neuroimaging and biosample collection in the Toronto adolescent and youth cohort study: rationale, methods, and early data. Biol Psychiatry Cogn Neurosci Neuroimaging. 2024;9(3):275–84. doi: 10.1016/j.bpsc.2023.10.013 37979944

[23] Quilty LC, Tempelaar W, Andrade BF, Kidd SA, Lunsky Y, Chen S. Cognition and educational achievement in the Toronto adolescent and youth cohort study: rationale, methods, and early data. Biol Psychiatry Cogn Neurosci Neuroimaging. 2024;9(3):265–74. doi: 10.1016/j.bpsc.2023.10.012 37979945

[24] Ancker JS, Benda NC, Reddy M, Unertl KM, Veinot T. Guidance for publishing qualitative research in informatics. J Am Med Inform Assoc. 2021;28(12):2743–8. doi: 10.1093/jamia/ocab195 34537840 PMC8633663

[25] Holtrop JS, Estabrooks PA, Gaglio B, Harden SM, Kessler RS, King DK, et al. Understanding and applying the RE-AIM framework: clarifications and resources. J Clin Transl Sci. 2021;5(1):e126. doi: 10.1017/cts.2021.789 34367671 PMC8327549

[26] IBM Corp. IBM SPSS Statistics for Mac, Version 31.0. Armonk (NY); 2025.

[27] Assarroudi A, Heshmati Nabavi F, Armat MR, Ebadi A, Vaismoradi M. Directed qualitative content analysis: the description and elaboration of its underpinning methods and data analysis process. J Res Nurs. 2018;23(1):42–55. doi: 10.1177/1744987117741667 34394406 PMC7932246

[28] Assarroudi A, Heshmati Nabavi F, Armat MR, Ebadi A, Vaismoradi M. Directed qualitative content analysis: the description and elaboration of its underpinning methods and data analysis process. J Res Nurs. 2018;23(1):42–55. doi: 10.1177/1744987117741667 34394406 PMC7932246

[29] QSR International Pty Ltd. NVivo qualitative data analysis software [Internet]. QSR International Pty Ltd; 2015 [cited 2024 Aug 20]. Available from: https://www.qsrinternational.com/nvivo/enabling-research/the-new-nvivo

[30] Nowell LS, Norris JM, White DE, Moules NJ. Thematic analysis: striving to meet the trustworthiness criteria. Int J Qual Methods. 2017;16(1):1609406917733847. doi: 10.1177/1609406917733847

[31] Johnson JL, Adkins D, Chauvin S. A review of the quality indicators of rigor in qualitative research. Am J Pharm Educ. 2020;84(1):7120. doi: 10.5688/ajpe7120 32292186 PMC7055404

[32] Frank L, Basch E, Selby JV, For the Patient-Centered Outcomes Research Institute. The PCORI perspective on patient-centered outcomes research. JAMA. 2014;312(15):1513–4. doi: 10.1001/jama.2014.11100 25167382

[33] American Academy of Family Physicians. Putting pre-visit planning into practice. Fam Pract Manag. 2015;22(6):34–8. https://www.aafp.org/pubs/fpm/issues/2015/1100/p34.html 26761083

[34] Dowding D, Merrill JA, Onorato N, Barrón Y, Rosati RJ, Russell D. The impact of home care nurses’ numeracy and graph literacy on comprehension of visual display information: implications for dashboard design. J Am Med Inform Assoc. 2018;25(2):175–82. doi: 10.1093/jamia/ocx042 28460091 PMC7647125

[35] Greenhalgh T, Wherton J, Papoutsi C, Lynch J, Hughes G, A’Court C, et al. Beyond adoption: a new framework for theorizing and evaluating nonadoption, abandonment, and challenges to the scale-up, spread, and sustainability of health and care technologies. J Med Internet Res. 2017;19(11):e367. doi: 10.2196/jmir.8775 29092808 PMC5688245

[36] Alexiuk M, Elgubtan H, Tangri N. Clinical decision support tools in the electronic medical record. Kidney Int Rep. 2023;9(1):29–38. doi: 10.1016/j.ekir.2023.10.019 38312784 PMC10831391

[37] Olakotan OO, Mohd Yusof M. The appropriateness of clinical decision support systems alerts in supporting clinical workflows: a systematic review. Health Inform J. 2021;27(2):14604582211007536. doi: 10.1177/14604582211007536 33853395

[38] Digital health: dashboards, dashboards, everywhere. Aust Prescr. 2024;47(2):46–7. doi: 10.18773/austprescr.2024.009 38737367 PMC11081738

[39] Branco D, Móteiro M, Bouça-Machado R, Miranda R, Reis T, Decoroso É, et al. Co-designing customizable clinical dashboards with multidisciplinary teams: bridging the gap in chronic disease care. Proceedings of the 2024 CHI Conference on Human Factors in Computing Systems [Internet]. New York, NY, USA: Association for Computing Machinery; 2024 [cited 2025 May 21]. p. 1–18. (CHI ’24). Available from: https://dl.acm.org/doi/10.1145/3613904.3642618 10.1145/3613904.3642618

[40] Sawyer SM, Azzopardi PS, Wickremarathne D, Patton GC. The age of adolescence. Lancet Child Adolesc Health. 2018;2(3):223–8. doi: 10.1016/S2352-4642(18)30022-1 30169257

[41] Nilsen P. Making sense of implementation theories, models and frameworks. Implement Sci. 2015;10:53. doi: 10.1186/s13012-015-0242-0 25895742 PMC4406164

[42] Helminski D, Sussman JB, Pfeiffer PN, Kokaly AN, Ranusch A, Renji AD. Development, implementation, and evaluation methods for dashboards in health care: scoping review. JMIR Med Inform. 2024;12:e59828. doi: 10.2196/59828 39656991 PMC11651422

[43] Fuller TE, Pong DD, Piniella N, Pardo M, Bessa N, Yoon C, et al. Interactive digital health tools to engage patients and caregivers in discharge preparation: implementation study. J Med Internet Res. 2020;22(4):e15573. doi: 10.2196/15573 32343248 PMC7218608

[44] Lewis CC, Boyd M, Puspitasari A, Navarro E, Howard J, Kassab H, et al. Implementing measurement-based care in behavioral health: a review. JAMA Psychiatry. 2019;76(3):324–35. doi: 10.1001/jamapsychiatry.2018.3329 30566197 PMC6584602

[45] Nasrallah C, Wilson C, Hamblin A, Young C, Jacobsohn L, Nakamura MC, et al. Using the technology acceptance model to assess clinician perceptions and experiences with a rheumatoid arthritis outcomes dashboard: qualitative study. BMC Med Inform Decis Mak. 2024;24(1):140. doi: 10.1186/s12911-024-02530-2 38802865 PMC11129391

[46] Powell BJ, Fernandez ME, Williams NJ, Aarons GA, Beidas RS, Lewis CC. Enhancing the impact of implementation strategies in healthcare: a research agenda. Front Public Health. 2019;7:3. doi: 10.3389/fpubh.2019.00003 30723713 PMC6350272

[47] Antonini M. An overview of co-design: advantages, challenges and perspectives of users’ involvement in the design process. J Des Think. 2021;2(1):45–60. doi: 10.22059/jdt.2020.272513.1018

[48] Strudwick G, Lo B, Kemp J, Jessa K, Tajirian T, White P, et al. Opportunities and challenges to enhance the value and uptake of Chief Nursing Informatics Officer (CNIO) Roles in Canada: a qualitative study. AMIA Annu Symp Proc. 2023;2022:1012–21. 37128401 PMC10148352

[49] Bird M, McGillion M, Chambers EM, Dix J, Fajardo CJ, Gilmour M, et al. A generative co-design framework for healthcare innovation: development and application of an end-user engagement framework. Res Involv Engagement. 2021;7(1):12. doi: 10.1186/s40900-021-00252-7 PMC7923456 33648588

[50] Zhuang M, Concannon D, Manley E. A framework for evaluating dashboards in healthcare. IEEE Trans Vis Comput Graph. 2022;28(4):1715–31. doi: 10.1109/TVCG.2022.3147154 35213306

[51] Tsangaris E, Edelen M, Means J, Gregorowitsch M, O’Gorman J, Pattanaik R, et al. User-centered design and agile development of a novel mobile health application and clinician dashboard to support the collection and reporting of patient-reported outcomes for breast cancer care. BMJ Surg Interv Health Technol. 2022;4(1):e000119. doi: 10.1136/bmjsit-2021-000119 35464815 PMC8987795

[52] Elston DM. Participation bias, self-selection bias, and response bias. J Am Acad Dermatol. 2021:S0190-9622(21)01129-4. doi: 10.1016/j.jaad.2021.06.025 34153389

