# Supplementary material for: An evaluation of the clinician-facing research dashboards from the Toronto Adolescent and Youth (TAY) Cohort Study in mental health care
Source: PLOS Ment Health. 2026 May 8;3(5):e0000605. doi: 10.1371/journal.pmen.0000605 (PMC13155665; doi:10.1371/journal.pmen.0000605)
Supplement: S1 Table — (DOCX) [file pmen.0000605.s001.docx]

**Supporting Information**

**S1 Table** Guidance for publishing qualitative research in informatics reporting checklist

| **Elements** | **Manuscript Location** |
| --- | --- |
| **Theory** |  |
| Cite theory appropriate to the topic being studied if applicable | Page 6, Lines: 79-90 |
| **Research question and study design** |  |
| State the research question | Page 7, Lines: 109-111 |
| State the study design and methodological perspective of the research | Page 8, Lines: 122-139 |
| **Sampling** |  |
| Describe the sampling approach | Page 10, Lines: 166-180 |
| Describe any approaches to ensure the inclusion of people from marginalized or underserved groups | N/A |
| Report and justify the sample size | N/A |
| If using saturation to determine sample size, report what type of saturation was used, and how saturation was assessed* | N/A |
| **Data collection** |  |
| Report how data were collected | Page 10, Lines: 166-169 & 173-180 |
| Report any methods for reducing bias in data collection and analysis* | Pages 13-14, Lines: 218-223 |
| **Data Analysis** |  |
| Describe data analysis methods, with appropriate citations |  |
| i. For deductive analysis, report how the theory was used in the data collection and analysis | Pages 13-14, Lines: 214-223 |
| ii. For inductive analysis, report how the steps of inductive analysis were done | N/A |
| iii. For theory development, report how categories were developed | N/A |
| Describe any methods for improving the dependability of coding | Pages 13-14, Lines: 214-223 |
| Report any measures for improving the credibility of findings or verifying interpretations | Pages 13-14, Lines: 214-223 |
| **Results** |  |
| Report sample size and characteristics of participants | Page 14, Lines: 226-232 |
| Support thematic findings with extracts, quotes, images, or observations | Pages 15-21, Lines: 259-382,  Table 1 & Figure 1 |
| Provide synthesis and interpretation | Pages 15-21, Lines: 259-382 |
| **Discussion** |  |
| Describe assumptions of the research and details of setting and context to illustrate transferability of findings | Pages 8-9, Lines: 140-164 &  Pages 22- 25, Lines: 417-481 |
| Describe relationship of findings, or new theory developed in the study, to existing theory | Page 21, Lines: 393-399 &  Page 25, Lines: 468-481 |
| Report limitations | Pages 27-28, Lines: 530-557 |
